# Motion-Based Confidence Score to Support the Practical Application of rPPG Methods in Health Monitoring

**DOI:** 10.1007/s10916-026-02412-2

**Published:** 2026-05-18

**Authors:** Miguel Arevalillo-Herráez, Yuyan Wu, Benjamin Tilbury, Naeem Ramzan

**Affiliations:** 1 Departament d’Informàtica, Universitat de València, Avda de la Universidad s/n, Burjassot, 46100 Valencia Spain; 2 Valencian Graduate School and Research Network of Artificial Intelligence (ValgrAI), Camí de Vera, s/n, Valencia, 46022 Spain; 3 School of Computing, Engineering and Physical Sciences, University of the West of Scotland, High Street, Paisley, PA1 2BE Renfrewshire UK

**Keywords:** rPPG, Heart rate estimation, Reliability scores

## Abstract

Remote photoplethysmography (rPPG) has gained popularity as a non-invasive technique for remote monitoring, as it can provide accurate measurements of an individual’s physiological signals under controlled conditions. However, the accuracy of rPPG can be affected by various factors, such as movement artifacts, changes in skin tone, and the presence of other sources of light in the environment. To improve the reliability of rPPG measurements in real-world monitoring settings and reduce the frequency of false alarms in health monitoring settings, we propose a confidence score indicating the quality of the predictions. This score was built by identifying meaningful variables related to motion that strongly correlate with the accuracy of the measurements and training a classifier with data coming from 3 distinct datasets, to improve the model’s robustness, reproducibility, and generalizability. Despite that only motion-related features have been considered, the high AUC values obtained in all cases were always above 0.93, demonstrating the model’s ability to detect inaccurate heart rate measurements.

## Introduction

Photoplethysmography (PPG) is a contact-based optical technique that measures blood volume changes in the skin to monitor cardiovascular activity [1, 2]. Remote photoplethysmography (rPPG) [3, 4] is the non-invasive version of this technology, which utilizes the changes in light reflectance or transmission from the skin to monitor vital signs remotely [5]. It can be used to detect changes in heart rate [6–8], blood pressure [9, 10], oxygen saturation [11, 12] and respiration rate [13, 14], as well as other indicators of health such as stress and fatigue [15]. rPPG is commonly used in a variety of settings, such as telemedicine, remote patient monitoring, and home-based health monitoring, as it allows healthcare professionals to continuously monitor the vital signs of their patients without the need for physical contact [16, 17].

Despite the many advantages associated with the non-contact nature of rPPG methods, there are several limitations to their applicability due to several factors that can significantly affect their accuracy. These are especially relevant in health monitoring settings, where the objective is to issue alerts when specific situations are detected. In these cases, motion and external light sources [18–20] may cause temporary artifacts that significantly affect the measurements, leading to frequent false-positive alarms that affect the practical usability of the method. In this kind of setting, the reliability of the measurement provided becomes critical.

In this study, we examined the impact of motion on the accuracy of rPPG measurements and developed a confidence score indicating the quality of the results. To focus on motion effects, we used several datasets in which other known sources of interference, such as significant changes in lighting, were scarce. We identified movement-related features that strongly correlated with the accuracy of the predictions and created a classification model that could accurately predict a confidence score for rPPG measurements. To ensure the model was robust and could generalize to different scenarios, we used data from 3 different datasets in a cross-validation setting and tested it using three commonly used signal extraction methods.

To the best of our knowledge, this is the first time in the literature that a confidence score that uses external factors such as the movement in the scene is suggested to support medical decisions and potential actions based on rPPG measurements. In this work, we propose several correlated motion-related features, and also provide a thoughtful evaluation of multiple classification approaches for this task, in an attempt to advance the applicability of existing rPPG methods for remote health monitoring.

The remainder of this paper is structured as follows: “Background and state of the art” section provides an overview of rPPG techniques, their application in heart rate estimation, and their relevance for remote patient monitoring. “Methodology” section details the methodology, including the selected features, datasets, development of the confidence score, and the performance metrics used for evaluation. “Results” section presents the experimental results. “Discussion” section offers a discussion of the findings and compares the proposed approach with existing methods. Finally, “Conclusions” section summarizes the main contributions and outlines directions for future work.

## Background and state of the art

Remote Photoplethysmography is based on the fact that the optical absorption of human skin varies with the amount of blood flowing through tissues with each heartbeat. Therefore, changes in the blood volume during the cardiac cycle result in subtle color changes on the skin, which are not visible to the naked eye but can be detected by optical sensors and used to measure vital signs. The human skin consists of three layers: the epidermis, dermis, and hypodermis. When exposed to light of a certain wavelength, the epidermis and dermis scatter the light, while the hypodermis diffuses it. rPPG measures the changes in the reflection of red, green, and blue light from the skin based on the contrast between specular and diffuse reflections. The increasing significance of rPPG methods is demonstrated by the numerous surveys that have been conducted on the topic [21–24]. This is supported by a wide range of applications, many of them in the area of remote patient monitoring.

Heart Rate (HR) measurement with rPPG requires a series of sequential steps that are organized into a processing pipeline, which consists of the following tasks: Selection of Region of Interest (ROI), artifact removal, Blood Volume Pulse (BVP) signal extraction and HR estimation. Selecting adequate ROIs is a challenging task that depends on the particular setting and may substantially affect the performance of rPPG methods. Facial regions are typically used, and this implies the use of face detection algorithms as part of the process. The full face, the forehead [25] or the cheeks [26] are some of the common choices adopted in the literature. The average pixel intensity within the ROI at each frame is used to generate a time series per available video channel. However, the intensities that form the time series are affected by factors not related to the HR, such as motion, illumination and/or shadows. To mitigate these effects, artifact removal and signal-denoising methods become necessary. The application of a moving average filter [27], together with normalisation and de-trending, is a common choice [28], but other combinations of filters and techniques have also been proposed [29]. The next step is extracting the BVP signal. The simplest option is to use the produced time series for one of the channels and discard the rest. The selected channel is usually green in the RGB case [30], but this method usually performs poorly because of its low robustness to motion. Other alternative approaches attempt to demix the filtered signals for each video channel. Some typical demixing methods include blind source separation methods, such as Independent Component Analysis (ICA) [31–34] or Principal Component Analysis (PCA) [35–38], but other techniques that are based on optical and/or physiological considerations have also been proposed. These include POS [39] and CHROM [40], which attempt to eliminate intensity and specular distortions that contain no pulse signal. Alternative techniques include Bayesian minimization [41], Independent Vector Analysis (IVA) [42], and other recent deep learning approaches [12, 43]. Finally, the resulting BVP signal is converted to the frequency domain, and the highest power response is used to compute the desired HR [44–46].

Despite the generally high accuracy of the rPPG methods in controlled settings [47], accuracy values reported in most works refer to an average deviation with respect to the true heart rates. While this measure is useful in evaluating a method, it does not necessarily provide an estimate of its potential effectiveness in patient monitoring applications. In these particular settings, the aim of rPPG is to detect and immediately report abnormal heart rates to remote medical services. This means that heart rate measurements need to be constantly taken, using data in a short time frame. In this specific context, the average deviations between real heart rates and values reported by rPPG are not that relevant, as the objective is to detect extreme values or variations that could be related to a series of medical conditions e.g. a heart stroke. In addition, some recent works show that the greatest deviations happen for extreme true heart rates [48] and false positives usually become more critical than false negatives. While the multiple constant measurements may convert the false positive into a small detection delay, an excessive amount of false negatives would make the method unusable because of the number of fake alerts that would be generated.

One possible approach already proposed in the literature is using anomaly detection mechanisms that can identify potentially erroneous measurements [49]. Nevertheless, while traditional anomaly detection methods may identify a significant increase in heart rate as an anomaly, they lack the capacity to determine whether this anomaly should raise an alert. A different approach is the use of a confidence score that indicates the reliability of the measurement and allows healthcare professionals to better determine the trustworthiness of the data. This, in turn, allows for more informed decision-making regarding the patient’s health and the appropriateness of issuing alerts. However, most available existing reliability measures depend on a reference signal that enables the use of error statistics such as mean absolute error (MAE), root mean square error (RMSE), and mean error rate percentage (MER), among others. These measures cannot be applied in realistic applications, where the reference signal is lacking. Several other previous studies have introduced reference-free quality assessment methods for rPPG signals. These approaches aim to estimate signal reliability without requiring ground-truth physiological measurements, relying instead on intrinsic properties of the extracted signal. Common strategies include computing self-scoring metrics such as the signal-to-noise ratio (SNR) [50], the average maximum cross-correlation between consecutive segments, or the relative difference of spectral peaks [51]. Other works have proposed combining ad hoc measures that exploit local temporal characteristics and global periodicity to derive a composite quality score [52].

More advanced approaches have leveraged deep learning techniques to estimate rPPG signal quality by learning complex representations directly from the signal [53, 54]. In these methods, deep neural networks are trained to process the raw or preprocessed rPPG waveform and output a quality index, bypassing the need for handcrafted metrics such as signal-to-noise ratio or spectral consistency. This data-driven strategy enables the model to capture subtle patterns and nonlinear relationships that traditional rule-based measures may overlook, potentially improving robustness across diverse conditions.

Also in the context of deep learning models, there have been more recent attempts to develop methods that provide quality scores by quantifying uncertainty in network predictions [55, 56]. Specifically, the total prediction uncertainty comprises both aleatoric and epistemic uncertainties, corresponding to data and model uncertainties, respectively [57, 58]. Aleatoric uncertainty originates from the intrinsic noise present in the recorded video data, while epistemic uncertainty results from the model’s lack of familiarity with previously unseen examples and can be reduced by acquiring additional training data.

**Fig. 1:**
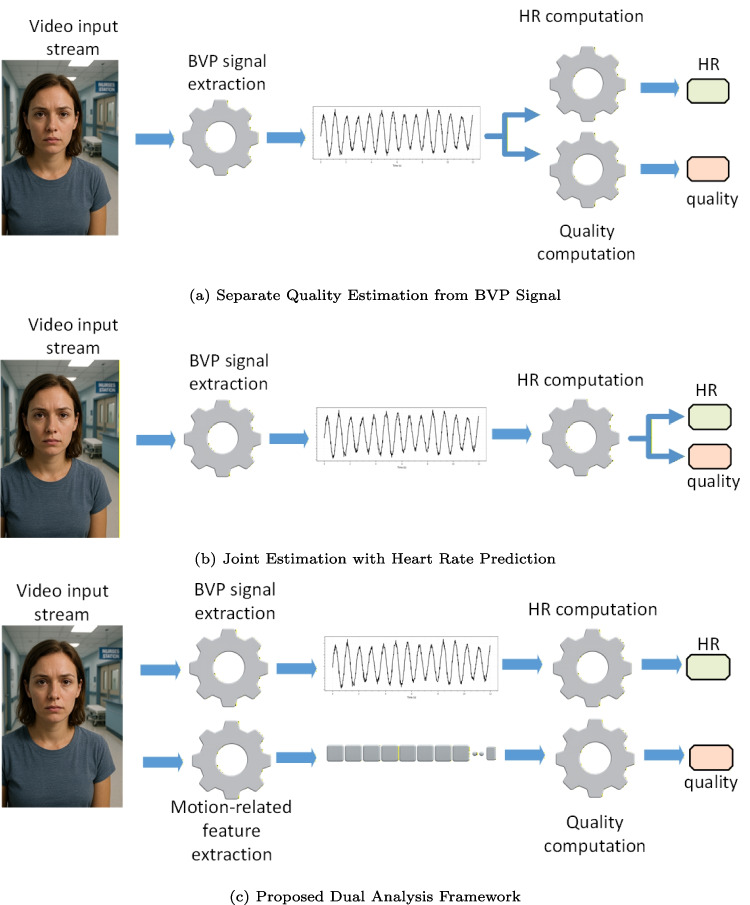
Comparison between the proposed approach and state-of-the-art methods. (**a**) Illustrates the traditional approach in which the quality index is computed independently from the extracted BVP signal. This estimation relies solely on the signal itself, either in its raw form or through handcrafted, signal-based metrics such as amplitude, periodicity, and noise characteristics. (**b**) Represents state-of-the-art methods that estimate quality jointly with heart rate prediction, often by modeling uncertainty in neural network outputs. These approaches still rely exclusively on the rPPG signal without considering contextual factors. (**c**) Depicts the proposed method, which combines BVP signal extraction with complementary motion analysis from the input video, addressing limitations of signal-only approaches

Despite the diversity of existing approaches, all current methods share a common limitation: they assess measurement quality exclusively from the BVP signal, ignoring external factors such as subject movement or illumination changes, which are well-documented to significantly affect accuracy. Figure 1 illustrates a conceptual comparison between state-of-the-art methodologies and the approach proposed in this work. Panels (a) and (b) represent existing strategies, where the quality index is ultimately derived from the BVP signal. In (a), quality estimation is performed as a separate process, while (b) depicts joint estimation with heart rate prediction, as commonly implemented when computing uncertainty in neural network outputs. Both approaches rely solely on signal morphology and derived parameters such as amplitude, periodicity, or noise characteristics, without considering contextual cues. In contrast, panel Fig. 1(c) illustrates our proposed framework, which introduces a dual analysis paradigm. While extracting the BVP signal, we simultaneously process the input video to derive motion-related features, which are subsequently utilized to compute the quality score. By incorporating motion descriptors, the proposed method accounts for dynamic conditions that traditional signal-only approaches disregard, thereby reducing the risk of overestimating quality in challenging real-world scenarios.

## Methodology

### Features

In order to understand the relationship between motion and the accuracy of rPPG measurements, we analyzed various indicators of motion. By doing so, we aimed to gather enough information to build a model that can accurately predict the reliability of rPPG measurements. The motion-related features we analyzed were Temporal Perceptual Information (*TI*), Face Motion on the x-axis ($FM_X$), Face Motion on the y-axis ($FM_X$), and Face Size Motion (*FSM*). To describe these features, let us assume a video window composed of a sequence of *n* frames $F_1, F_2 \ldots F_n$.

#### Temporal Perceptual Information

(*TI*) is a commonly used metric to approximate the motion of a scene, and it is defined in the ITU-T Recommendation P.910 “Subjective video quality assessment methods for multimedia applications” [59]. It is calculated from motion differences between successive frames as

1 $$\begin{aligned} TI=\max _{i=2 \ldots n}\sigma [M_i], \end{aligned}$$

where $\sigma$ stands for the standard deviation and $M_i(x,y)=L_i(x,y)-L_{i-1}(x,y)$, with $L_i(x,y)$ representing the luminance value of pixel at coordinates (*x*, *y*).

#### Face-Motion along the X- and Y-axes

($FM_X$ and $FM_Y$) are computed as the average displacement along the x- and y- axes of the *l* landmark points in the face model used to define the ROI, using the expressions

2 $$\begin{aligned} \frac{1}{l \cdot (n-1) } \sum _{i=1}^{l} \sum _{j=2}^{n} D^{x}_{ij} \end{aligned}$$

and

3 $$\begin{aligned} \frac{1}{l \cdot (n-1)} \sum _{i=1}^{l} \sum _{j=2}^{n} D^{y}_{ij}, \end{aligned}$$

where $D^{x}_{ij}$ and $D^{y}_{ij}$ are the absolute value of the displacement of landmark point *i* from frame $F_{j-1}$ to frame $F_{j}$, along the x- and y- axes, respectively.

#### Face Size Motion

(*FSM*) controls displacements along the z- axis, and is computed as

4 $$\begin{aligned} \frac{1}{n-1} \sum _{i=2}^{n} |N_i-N_{i-1}|, \end{aligned}$$

where $N_i$ is the number of pixels within the ROI of frame $F_i$ and $|\cdot |$ is the absolute value of the argument.

The computation of these features is relatively simple and does not affect the real-time operation of the rPPG pipeline, as it requires less than one-twentieth of the total pipeline processing time.

### Databases

In order to ensure the validity of the results across different setting and conditions, all our experiments were conducted on 3 independent uncompressed databases. We used the 3 following publicly available data sets in our study: UBFC-RPPG [60], LGI-PPGI-Face-VideoDatabase (LGI-PPGI-FVD) [61] and Pulse Rate Detection Dataset (PURE) [62], which are described below. Other databases commonly used in the literature were not included in the analysis because they are provided in compressed format, e.g. COHFACE [63], V4V [64] or MAHNOB-HCI [65], introducing a potential and uncontrolled source of inaccuracies in the signal.

#### UBFC-RPPG

[60] consists of 50 PPG and video recordings of 49 subjects. The videos were recorded at 30 fps with a resolution of $640 \times 480$ in 8-bit RGB format using a low cost webcam (Logitech C920 HD Pro) placed at a distance of 1 meter from the subjects. 42 videos feature subjects playing a time-sensitive mathematical game aimed at varying their heart rate to emulate a scenario of normal activity, while the remaining 8 are simple scenarios of the subjects sitting still. Ground truth heart rates were obtained by using a synchronized pulse oximeter finger clip sensor.

#### LGI-PPGI-FVD

[61] contains 24 video recordings of 6 subjects during four different sessions (resting with no intended head motion, head and facial motion, exercising on a cycle ergometer in a gym with no set restrictions, and talking in a real-world urban environment). Videos were captured at 25 fps with a resolution of $640 \times 480$, in uncompressed format. Pulse oximetry recordings are also provided as ground truth data, captured at 60 Hz; but they are missing for one session for one user. We only used the 5 users that had all sessions available (20 videos for 5 subjects). This dataset contains some challenging scenarios with motion in real-world situations, including the presence of multiple subject and uncontrolled lighting conditions.

#### PURE

[62] contains 59 video recordings of 10 subjects in sitting position during six different controlled scenarios (resting with no intended head motion, talking, slow head translation of 7% of the face height per second, fast head translation of 14% of the face height per second, small head rotation of approximately 20 degrees, and large head rotation of approximately 35 degrees). All videos were captured using a ec0274CVGE camera by SVS-Vistek GmbH at 30 fps with a resolution of $640 \times 480$, in lossless compressed format. Ground truth data is provided in the form of reference pulse waveform signals recorded with a pulox CMS50E finger clip pulse oximeter at a 60 Hz sampling rate.

**Table 1:** Summary of characteristics of the three databases considered in this work

Dataset	# Subjects	# Videos	Duration (Average)	FPS (Average)	Resolution (Average)
UBFC-RPPG [66]	49	50	68s	29.02	$640 \times 480$
LGI-PPGI [67]	5	20	135s	25.00	$640 \times 480$
PURE [68]	10	59	116s	29.97	$640 \times 480$

The three databases used in this study included different levels of motion based on the specific scenario being recorded. In some specific scenarios, slight changes in lighting conditions were also observed, but they were not significant. For example, the lighting condition in PURE was daylight through a large window frontal to the face with clouds slightly changing illumination over time; and the fourth session in LGI-PPGI-FVD was recorded during an urban conversation including natural varying illumination conditions in addition to head and facial motion. Table 1 summarizes the main characteristics of each dataset described above.

### HR computation

To ease the replication of experiments, we used a typical rPPG pipeline in our experiments. This pipeline was defined in phuselab^1^ as a framework to study, develop and compare new rPPG methods in a principled and reproducible way. We adopted it as our test bed as the full code is available under a GPL-3.0 license, easing experimental replication. The HR at every second was computed by considering scenes composed of all frames in the immediately preceding 12-second window, and the ROI at each frame was determined by extracting facial keypoints by using Blazeface, a fast and light-weight face detector from Google Research tailored for mobile GPU inference [69]. These keypoints were then used to compute both convex hulls of the face region and also of non-skin parts, such as the mouth and the eyes, which were subtracted from the ROI. Pixels values within the ROI were then averaged independently for each of the 3 channels, to produce one time series per channel, containing the average values for all frames in the 12 s window. To allow for a fair comparison, we did not apply any artifact removal or signal-denoising algorithms.

In order to fairly evaluate the motion effect under a variety of different settings, 3 different BVP signal extraction methods were attempted. As a first option, we considered using the green channel only, as this method is usually taken as a baseline [30]. We also considered POS [39] and CHROM [40], as the most commonly used methods due to their higher accuracy because of their ability to mitigate intensity and specular distortions that contain no pulse signal.

**Fig. 2:**
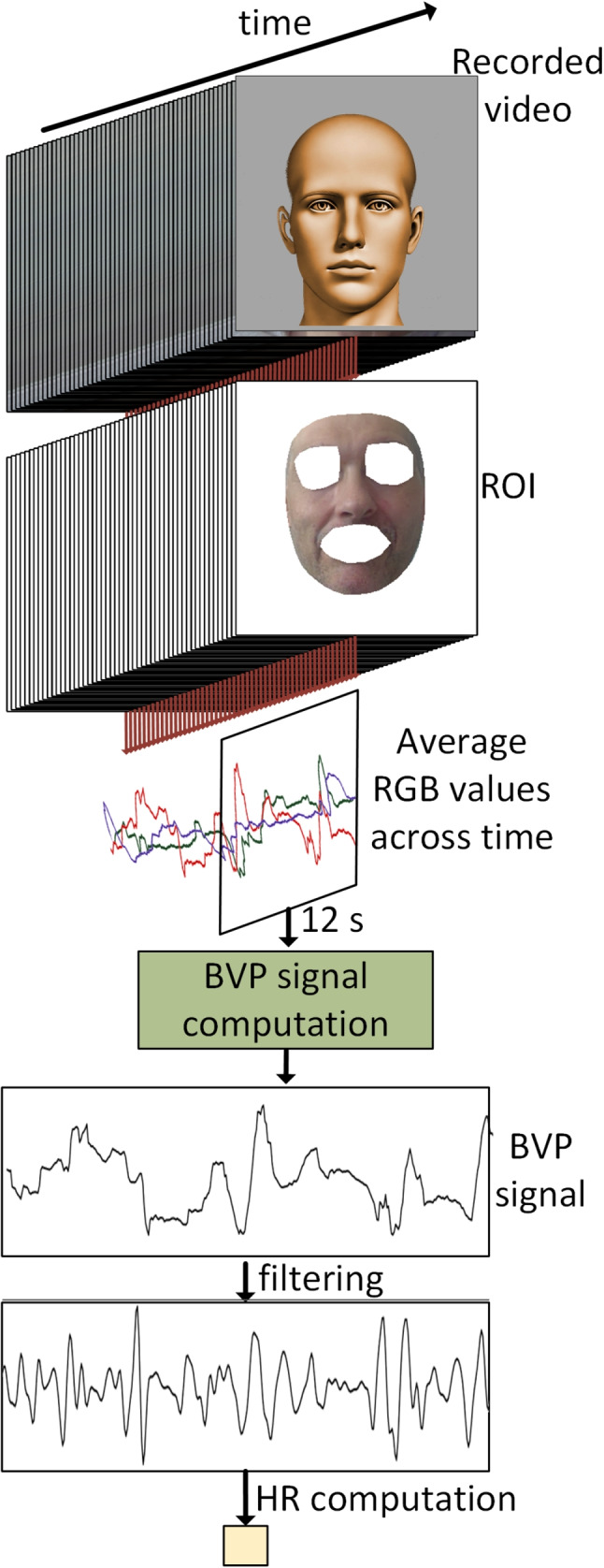
HR Computation, computed at every second

Finally, the computation of the HR proceeded by first applying a Butterworth bandpass filter followed by a Hann window. Then, the spectral density was calculated and the HR was obtained by quadratic peak interpolation. Figure 2 summarizes the main steps performed to test each alternative. The approaches evaluated differ in the BVP signal computation step, which has been colored in green.

### Construction of confidence score

The four features described above in “Features” section were used to build a classifier. The target variable was the reliability of the measurement. To do this, we transformed the data into a binary classification problem. Measurements with an absolute error below 2 heartbeats per second were considered reliable (class 1) while those with an absolute greater than 6 were considered non-reliable (class 0). Samples with an absolute error outside these ranges were considered undefined and were not used neither for training nor testing the models. This labeling strategy avoids the artificial discontinuities that would arise from using a single hard threshold to separate reliable from unreliable samples. Such a thresholding approach would make the classification overly sensitive to minor fluctuations or noise, since samples with nearly identical errors, such as 1.99 bpm and 2.01 bpm, would be assigned to different classes despite being practically indistinguishable. As measurements falling within the intermediate 2–6 bpm range do not provide a clear indication of whether the underlying estimation should be considered “reliable” or “unreliable”, excluding this ambiguous region allows the classifier to be trained only on cases where the distinction between classes is meaningful and well defined. While these specific thresholds are not tied to a formal standard, they reflect practical interpretations of acceptable accuracy levels.

To evaluate the model’s robustness, we conducted a 5-fold cross-validation using data coming from the 3 datasets described in “Databases” section. In each run, we used data in one fold as the test set and data in the remaining four folds for training. The validation set was extracted from the training data to fine-tune the model’s parameters when appropriate. In all experiments, we applied subject-level stratification to ensure that all samples from a given participant were assigned to the same fold. The same procedure was followed when generating validation sets. This strategy prevents the model from exploiting subject-specific patterns, which could artificially inflate performance and limit its ability to generalize to unseen individuals.

To better understand the nature of the problem and identify the most appropriate classifier for the problem at hand, we evaluated multiple classification approaches, namely: Fine tree, Linear Discriminant Analysis (LDA), Logistic Regression, Naive Bayes assuming features are normally distributed, Linear Support Vector Machine (SVM), SVM with Gaussian kernel, K-nearest neighbour (k-NN) with k=1, and a neural network with 3 layers of 10 neurons and ReLU activation. Two ensemble methods based on decision trees were also included in the comparison, namely Boosted Trees and Bagged trees. The available implementations of MATLAB version R2021a were used in all the classification experiments. For the fine tree, we set the maximum splits to 100 and used Gini’s diversity index as the split criterion. We used automatic hyperparameter optimization for the SVM classifiers. For the Boosted Trees, we utilized AdaBoost with a maximum of 20 splits, 30 learners, and a learning rate of 0.1. In the case of the Bagged Trees method, we configured the Random Forest with 9 maximum splits and 30 learners.

### Classification performance metrics

Our classifier is designed to output a continuous reliability score rather than a binary label. This score can then be interpreted or thresholded by downstream systems according to the requirements of a specific application by selecting the operating points that best match their needs. Metrics such as precision, recall, or F1-score require selecting a specific decision threshold to convert this continuous score into a binary label (“reliable” vs. “unreliable”). However, choosing such a threshold is inherently arbitrary and would strongly influence the reported values of these metrics. Because the threshold is not fixed by the application nor by any established standard, different threshold choices would yield very different precision/recall/F1 values, making the comparison potentially misleading. In contrast, the ROC (Receiver Operating Characteristic) curve and the corresponding AUC (Area Under the Curve) summarize the classifier’s behavior across all possible thresholds, providing a threshold-independent evaluation. This is particularly important in our context for two reasons. First, due to the class imbalance, threshold-dependent metrics may give an overly optimistic or pessimistic view depending on where the threshold is placed. Second, the operational meaning of what constitutes an acceptable reliability threshold is application-dependent: medical monitoring systems, fitness devices, or research-grade measurements may each require different trade-offs depending on the impact of false alarms and missed detections. By reporting ROC–AUC, we provide a performance measure that is agnostic to these application-specific constraints while still capturing the classifier’s ability to discriminate reliably across the full range of its output scores. The AUC looks at the whole range of possible thresholds and evaluates the ability of the classifier to rank instances correctly across all threshold values, taking into account the overall ordering of predicted scores for the positive and negative instances to provide a single threshold-independent summary score. In this way, the AUC is a measure of the trade-off between the true positive rate (sensitivity) and the false positive rate (specificity) and gives the probability that a classifier will rank a randomly chosen positive instance higher than a randomly chosen negative instance. Therefore, a model with an AUC score of 1 is considered a perfect classifier, while a model with an AUC-ROC score of 0.5 is considered no better than random guessing.

**Table 2:** Number of reliable, unreliable, and discarded samples in each database, for the 3 BVP methods considered. Groups of columns correspond to datasets, and rows to BVP signal-extraction methods

	UBFC-RPPG			LGI-PPGI-FVD			PURE			Global		
	Reliable samples	Unreliable samples	Discarded samples	Reliable samples	Unreliable samples	Discarded samples	Reliable samples	Unreliable samples	Discarded samples	Reliable samples	Unreliable samples	Discarded samples
CHROM	2507	93	222	1450	761	243	3196	51	121	7153	905	586
POS	2536	58	228	1834	371	249	3233	25	110	7603	454	587
GREEN	2347	196	279	1132	1082	240	3011	199	158	6490	1477	677

The distribution of reliable, unreliable, and discarded samples shown in Table 2 provides a useful estimate with regard to the reliability of the 3 BVP signal extraction methods considered in the paper, at the same time as illustrates the extent of the class imbalance. Table 3 shows the same data but in terms of the percentage of unreliable samples over the set of all measurements considered in each database. As can be observed, POS is the best-behaving signal extraction method in all datasets, followed by CHROM with nearly double the number of unreliable measurements. Lastly, the green channel is the least effective option among the three, resulting in significantly higher rates of inaccurate samples compared to the other two methods. This can be attributed to the fact that the green channel may not be as robust in capturing the necessary information for HR measurement, especially in the presence of motion and other artifacts. This highlights the importance of carefully choosing the appropriate method and channel for HR measurement to ensure accurate and reliable results.

Table 3 also provides a feeling about the magnitude of the problems at hand. For example, LGI-PPGI-FVD contains high rates of unreliable measurements, which range from 16.8% of the samples considered when using POS to 48.9% when using the green channel. This is because this database contains specific scenarios with motion that significantly affect HR measurement in a large percentage of samples, unlike other datasets with a larger proportion of still scenes. Such scenarios containing motion are similar to the ones found in realistic remote patient monitoring situations. The high rates of unreliable measurements found in this database would indeed be problematic for real-world remote monitoring scenarios, as it would result in a large number of false alerts. Our proposed confidence score helps determine which alarms are legitimate and which are not, mitigating the impact of false positives when monitoring vital signs.

**Table 3:** Percentage of considered measurements classified as unreliable for each dataset–method combination. Columns correspond to datasets, and rows to BVP signal-extraction methods

	UBFC-RPPG	LGI-PPGI-FVD	PURE	Total
CHROM	3.6%	34.4%	1.6%	11.2%
POS	2.2%	16.8%	0.8%	5.6%
GREEN	7.7%	48.9%	6.2%	18.6%

## Results

### Feature correlation analysis

When designing a classifier, one of the most important considerations is the correlation of the features being analyzed with the variable of interest, as this is related to whether the feature contains information that is relevant to the variable and can be used to discriminate. Correlation analysis was performed for each motion feature in each of the databases, by computing the Pearson Correlation Coefficient (PCC) between each feature and the absolute measurement error in the rPPG prediction. By analyzing the correlation between the features and the absolute error, we were able to identify the variables that had a relevant impact on the accuracy of the HR measurements.

The PCC $r_{x,y}$ is a measure of the strength and direction of the linear relationship between two variables and is defined as the ratio between the covariance of two variables and the product of their standard deviations, i.e.

5 $$\begin{aligned} r_{x,y}=\frac{\sum _{i=1}^{n}(x_i-\overline{x})(y_i-\overline{y})}{\sqrt{\sum _{i=1}^{n}(x_i-\overline{x})^2\sum _{i=1}^{n}(y_i-\overline{y})^2}} \end{aligned}$$

where $x_i$ are the values of the x-variable in a sample, $\overline{x}$ is the mean of the values of the x-variable, $y_i$ are the values of the y-variable in a sample and $\overline{y}$ is the mean of the values of the y-variable. A value of 1 indicates a strong positive correlation, a value of -1 indicates a strong negative correlation, and a value of 0 indicates no correlation.

To measure the correlations between each feature described in “Features” section and the absolute heart rate error, we considered a value for each HR computed according to the procedure described in “HR computation” section. For each 12-second sliding window, we calculated the absolute error of the estimated heart rate and the corresponding value of the feature using the formulae described in “Features” section. This resulted in a value pair $(x_i,y_i)$ for each window, where $x_i$ represents the absolute error in the measurement and $y_i$ represents the value of the feature.

The PCC *r* between the proposed features and the absolute error on the heart rate measurement are reported in Table 4. P-values were $<10^{-40}$ in all cases. The moderate correlation between the features and the measurement error suggests that the features are strongly related to the variable of interest and are likely to be useful in predicting it.

**Table 4:** Pearson correlation coefficient between each computed motion feature and the absolute measurement error in the rPPG prediction

Feature	r
*TI*	0.594
$FM_X$	0.512
$FM_Y$	0.546
*FSM*	0.374

**Fig. 3:**
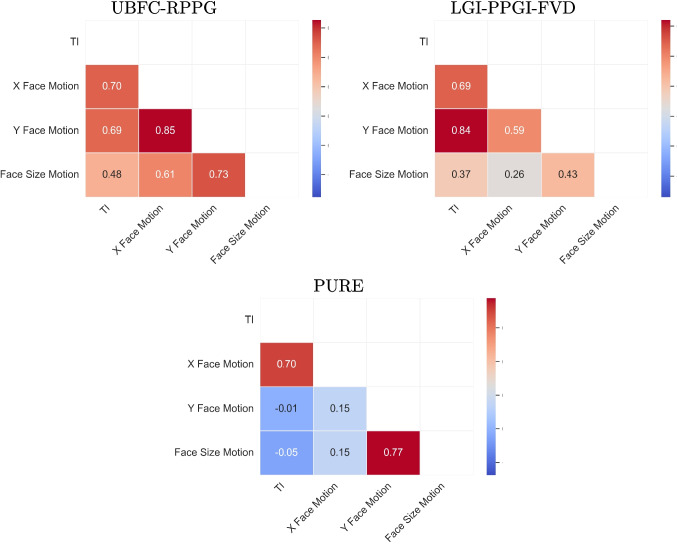
Correlation matrices of the four motion features across the three datasets considered in this study. Each subplot corresponds to a different dataset

An additional aspect worth analyzing is the correlation among the extracted features, as this provides insight into potential redundancy and complementarity between them. Figure 3 presents heatmaps illustrating these correlations across the three datasets considered. As shown, the degree of correlation between the four features is highly dependent on the dataset and is not uniformly strong across all cases, although certain pairs exhibit high correlation in specific scenarios. For instance, while TI might intuitively be expected to correlate with other motion-related features, this assumption does not always hold. In situations where scene motion occurs predominantly in the background or is constrained to a single dimension, such as in the PURE dataset, the correlation between TI and other motion descriptors becomes even negative. Furthermore, the nature of movement varies significantly depending on the recording conditions and the type of actions performed, which introduces substantial variability across datasets.

These observations suggest that each feature captures distinct aspects of motion and perceptual dynamics. Consequently, considering all four features simultaneously is likely to provide a richer and more comprehensive representation of the underlying phenomena. This multi-feature approach can enhance the robustness and accuracy of predictive models or estimation frameworks, as it leverages complementary information rather than relying on a single dominant descriptor.

### Evaluation of confidence score

We have evaluated the confidence scores produced by the system in the three datasets considered, repeating the experiment for the three different BVP signal extraction methods (CHROM, POS and green channel). Results are shown in Tables 5, 6 and 7, respectively, in terms of AUC. To ease the comparison, the best results in each database have been marked in bold, and AUCs for the best classification algorithm in each case have been reported in the last row of each table.

**Table 5:** AUC scores across the three datasets when using CHROM for BVP signal extraction and various classifiers. Each row corresponds to one classifier, and each column to one dataset

	CHROM		
Classifier	UBFC-RPPG	LGI-PPGI-FVD	PURE
Fine tree	0.68	0.84	0.80
LDA	0.67	0.81	0.74
Logistic regression	0.70	0.81	0.74
Naive Bayes	0.59	0.80	0.73
Linear SVM	0.79	0.80	0.53
Gaussian SVM	0.71	0.88	0.86
K-NN	0.73	0.83	0.76
Boosted trees	**0.81**	0.88	0.87
Bagged trees	**0.81**	**0.93**	**0.90**
Neural network	**0.81**	0.89	0.87
Best	0.81	0.93	0.90

**Table 6:** AUC scores across the three datasets when using POS for BVP signal extraction and various classifiers. Each row corresponds to one classifier, and each column to one dataset

	POS		
Classifier	UBFC-RPPG	LGI-PPGI-FVD	PURE
Fine tree	0.71	0.83	0.71
LDA	0.66	0.79	0.59
Logistic regression	0.68	0.79	0.62
Naive Bayes	0.61	0.74	0.73
Linear SVM	0.55	0.76	0.52
Gaussian SVM	0.71	0.88	0.87
K-NN	0.71	0.84	0.68
Boosted trees	0.75	0.90	0.75
Bagged trees	**0.81**	**0.93**	**0.88**
Neural network	0.79	0.91	0.85
Best	0.81	0.93	0.88

**Table 7:** AUC scores across the three datasets when using green channel information only and various classifiers. Each row corresponds to one classifier, and each column to one dataset

	Green channel		
Classifier	UBFC-RPPG	LGI-PPGI-FVD	PURE
Fine tree	0.79	0.89	0.86
LDA	0.78	0.82	0.83
Logistic regression	0.78	0.82	0.83
Naive Bayes	0.75	0.81	0.83
Linear SVM	0.73	0.83	0.93
Gaussian SVM	0.86	0.90	0.93
K-NN	0.78	0.89	0.89
Boosted trees	0.87	0.90	0.93
Bagged trees	**0.89**	**0.94**	**0.96**
Neural network	0.85	0.90	0.95
Best	0.89	0.94	0.96

**Fig. 4:**
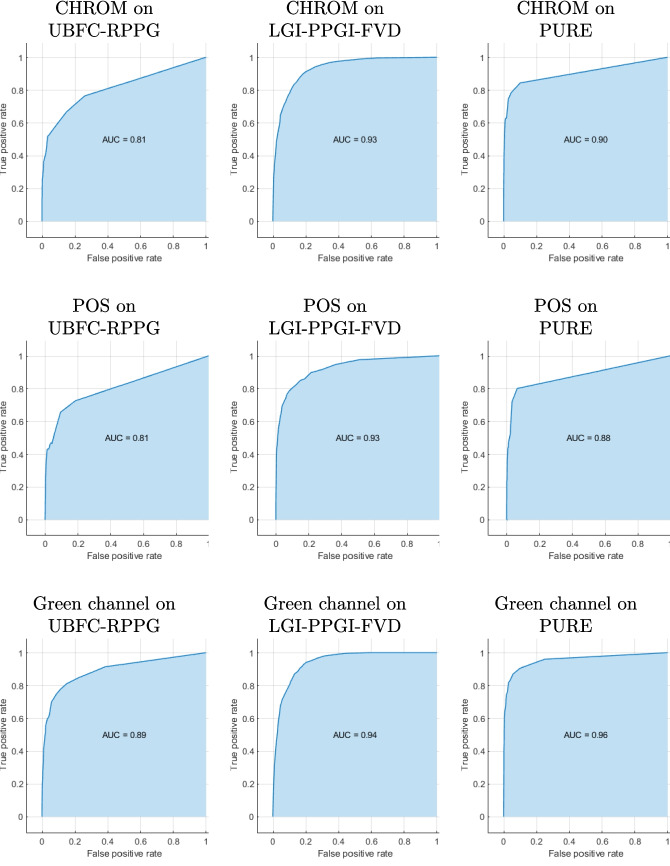
ROC curves and corresponding AUC values for the various BVP signal-extraction methods across the three evaluated datasets. Each column corresponds to a different dataset, while each row represents a different extraction method

For completeness reasons, we provide plots for the ROC curves and AUCs values for the Bagged trees classifier in Fig. 4, which shows the best results when the method is applied on each of the three different datasets.

In general, significantly worse results were obtained in the UBFC database. This could be due to the fact that this database only included one video per user, which meant that the classification algorithm did not have the opportunity to identify and learn patterns that are unique to each individual.

With regard to results as a function of the BVP signal extraction method, the use of the green channel yielded slightly better AUCs than POS and CHROM. This is a consequence of the lower accuracy of this method, which leads to a more balanced classification problem. However, the better AUCs did not outweigh the far larger number of inaccurate measurements produced by the method, as compared to POS, which appears to be a far more reliable technique according to the data presented in Table 3. As can be observed in this table, the use of the green channel produced over triple the number of unreliable measurements than POS.

In relation to the performance of classification algorithms, bagged tree ensembles consistently led to notably better performance than the rest. This consistency is somehow surprising, and it happened across all different BVP signal extraction methods and datasets. Overall, the high AUCs obtained in all cases support the ability of the model to accurately detect inaccurate heart rate measurements.

### Generalization of the approach

The final set of experiments aims to evaluate the generalization capability of the proposed method, ensuring that it does not overfit to the training data. These experiments are designed to verify that the method can learn patterns applicable across various contexts and maintain strong performance on unseen data, extending beyond the specific examples used during training.

We test the method generalization using two complementary approaches. First, we perform a cross-dataset experiment in which models are trained on data from two datasets and subsequently evaluated on an entirely different dataset. Second, we analyze the effect of using combined data from all three datasets simultaneously. In both experiments, we maintained the 5-fold cross-validation setting that was used for the evaluation of the confidence score, always ensuring that no data was simultaneously included in the training and test sets.

Table 8 presents the results from the cross-dataset experiment when using the bagged trees classifier, which was the one showing the best performance in the previous experiment. For each dataset tested, the models were trained using all data contained in the remaining two datasets. As expected, the results from the experiments described in this section show that results improve when the data used from training comes from exactly the same setting. However, the slight performance reduction when compared to the results shown in Tables 5, 6 and 7 still reinforce the generalizability of the proposed method.

**Table 8:** AUC scores for the cross-dataset experiment. Each column reports the AUC values for one signal-extraction method when testing on the dataset indicated in the first column and training on the remaining two datasets

	CHROM	POS	Green channel
UBFC-RPPG	0.79	0.78	0.82
LGI-PPGI-FVD	0.87	0.80	0.86
PURE	0.82	0.85	0.85

**Fig. 5:**
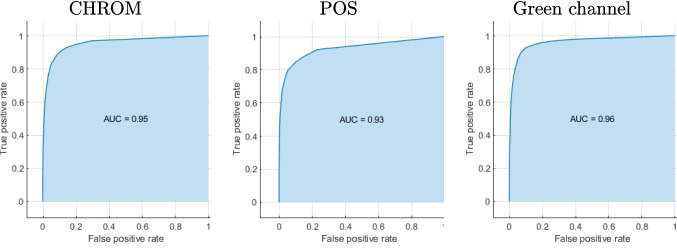
ROC curves and corresponding AUC values for the various BVP signal-extraction methods, obtained using the full available dataset and the best-performing classifier identified in Table 9 (Bagged Trees)

The results obtained by combining samples from all datasets in both the training and testing phases are presented in Table 9. Figure 5 shows the plots for the ROC curves and AUCs values for the best-behaving classifier (Bagged trees), for each different signal extraction method.

**Table 9:** AUC results when simultaneously considering the data from the 3 databases, when using different BVP signal extraction methods and classifiers

	CHROM		
Classifier	CHROM	POS	Green channel
Fine tree	0.90	0.84	0.90
LDA	0.88	0.85	0.87
Logistic regression	0.89	0.85	0.88
Naive Bayes	0.88	0.84	0.85
Linear SVM	0.86	0.82	0.85
Gaussian SVM	0.89	0.87	0.91
K-NN	0.88	0.84	0.89
Boosted trees	0.94	0.92	0.93
Bagged trees	**0.95**	**0.93**	**0.96**
Neural network	0.93	0.91	0.94
Best	0.95	0.93	0.96

As can be observed, there is a consistent improvement across all cases, indicating that the best outcomes were achieved when data from all databases were integrated. This observation holds true regardless of the BVP signal extraction method employed, suggesting that larger and more diverse datasets contribute to the generation of more reliable confidence scores. In particular, AUCs achieved when using the bagged trees approach on the full set of data were above 0.93 in all cases, indicating the high performance of the produced models in discriminating between accurate and inaccurate measurements. These findings imply that further enhancements may be possible by expanding the amount of training data used in the model.

## Discussion

The integration of contextual information into rPPG quality assessment represents a significant advancement toward improving robustness in real-world applications. By using motion-related features extracted from the input video, our approach addresses a key limitation of traditional signal-only methods, which fail to account for external factors such as subject movement or illumination changes. Table 10 summarizes the key differences between the proposed dual-analysis framework and existing state-of-the-art approaches, according to the classification of methods offered in Fig. 1. This table highlights distinctions in input signals, quality estimation strategies, contextual awareness, and expected performance under real-world conditions.

**Table 10:** Comparison between state-of-the-art rPPG quality assessment approaches and the proposed dual analysis framework

	Separate quality from BVP	Joint with HR	Proposed dual analysis
Input signals	BVP only	BVP only	BVP + Video frames
Quality estimation strategy	From signal or specific metrics (SNR, amplitude, periodicity, spectral peak consistency, cross-correlation of segments)	By quantifying uncertainty in network predictions when computing HR estimates	Supervised estimation using motion descriptors
Context considered	None (BVP signal-only)	None (BVP signal-only)	Explicit motion features from the video
Strengths	Simple, low compute, interpretable metrics	Integrates naturally with neural pipelines	More robust in unconstrained settings; reduces overconfidence under motion; improves generalization
Limitations	Highly sensitive to motion	Highly sensitive to motion	Additional video processing cost for independent computation of reliability metric
Expected behavior in real-world scenarios	Unstable reliability; frequent false positives/negatives	Moderate, depends on HR model robustness	Lower false confidence under motion
Computational cost	Depends on the particular method, but generally low-moderate	Moderate (uncertainty computation). Depends on the particular HR estimation method, but generally moderate. Require uncertainty computation	Moderate–High (due to real-time motion feature extraction)

Experimental results across three datasets and multiple signal extraction methods demonstrate that the dual analysis framework effectively identifies inaccurate measurements and exhibits strong generalization capability. The best performance was achieved using a bagged trees ensemble, particularly when applied to rPPG signals extracted with the POS method, which minimizes the number of unreliable measurements.

However, this study also has limitations. All datasets were recorded in controlled environments, which may not fully capture the complexity and variability of real-world conditions. Besides, while this work focuses on motion as a primary source of rPPG quality degradation, we acknowledge that other factors, such as skin tone and lighting, can also substantially affect signal quality and algorithmic performance. Prior studies have shown that differences in melanin concentration influence the amount of light absorbed and reflected by the skin, thereby impacting the strength and stability of the photoplethysmographic signal [70]. However, the datasets used in our experiments do not provide reliable or sufficiently diverse annotations for skin pigmentation, making it infeasible to perform a systematic analysis of skin-tone effects within the scope of this study. Regarding lighting, sudden illumination changes or low-light conditions can further introduce noise and reduce signal-to-noise ratio, thereby degrading the accuracy of both the signal-extraction methods and the downstream reliability assessment. Unfortunately, the datasets employed here were neither specifically designed to explore lighting-related variability, and illumination conditions remain relatively homogeneous or uncontrolled, preventing a rigorous evaluation of how lighting affects reliability predictions. Future research should validate the proposed approach in unconstrained settings, incorporating diverse scenarios that reflect typical variations in lighting, skin tone, and motion. Additionally, expanding the size and diversity of training data could further improve model robustness. Designing representative features capable of capturing major changes in environmental conditions will be essential for developing a confidence score that accounts for multiple sources of noise.

Another significant limitation of our study relates to the use of the phuselab pipeline throughout our research. While this approach provided a foundation for our analysis, more recent and advanced pipelines, such as Face2PPG [71], offer faster and more effective alternatives. Moreover, unsupervised signal extraction methods like Contrast-Phys [72], which do not rely on ground truth signals for training, represent a substantial advancement in the field. The reliance on the phuselab pipeline eases experimental replication of the study but also restricts its scope and diminishes the potential impact of the proposed confidence score, particularly in identifying issues with heart rate estimation when using these more sophisticated methods.

The new paradigm also presents opportunities for further improvements. Future work could explore multimodal fusion strategies by integrating additional contextual cues such as illumination metrics or facial landmark stability. Leveraging deep learning architectures capable of jointly modeling temporal and spatial dependencies may enable adaptive weighting of signal and contextual features, improving generalization across diverse environments. Ultimately, this approach aligns with the growing demand for unconstrained remote monitoring solutions, with potential applications in telemedicine, fitness tracking, and driver state assessment.

Finally, it is also essential to continue exploring strategies that focus on the effective application of the proposed confidence score. Specifically, responses to detected heart rates that fall outside of safe limits should be carefully crafted to strike a balance between minimizing the risk of delayed reactions and avoiding excessive or unnecessary alarms. One potential strategy that could be considered is the implementation of adaptive video-capturing techniques that adjust the frame rate based on the confidence score. For example, when low confidence scores are detected, the capturing rate could be increased to collect more data to improve the system’s reliability. This would allow for a more dynamic and responsive monitoring system that can adapt to noisy data in real-time. Another alternative mechanism consists of using the confidence score to weigh the importance of the alarm, or triggering them only when multiple predictions with a high level of confidence agree on the measurement, by establishing appropriate thresholds that strike an adequate balance between accuracy and delay in detection.

## Conclusions

This work addressed the challenge of improving the reliability of remote photoplethysmography (rPPG) measurements under motion by introducing a confidence score based on contextual information. Unlike existing approaches that assess quality solely from the rPPG signal, our method uses motion-related features extracted from the input video, providing a robust estimation of measurement reliability. Experimental results obtained across three datasets and multiple signal-extraction methods demonstrate that the proposed dual-analysis framework effectively identifies inaccurate measurements and shows consistent performance when applied to unseen data.

The proposed approach has the potential to enhance the applicability of rPPG in real-world scenarios such as health monitoring, where dynamic conditions are common. Future work will focus on validating the method in unconstrained environments, incorporating additional contextual cues such as illumination changes, and exploring adaptive strategies for real-time monitoring. These steps will enable the development of more reliable and scalable rPPG systems capable of operating effectively in diverse conditions.

## Data Availability

The datasets used and/or analyzed during the current study are publicly available datasets and can be requested from their authors.

## Footnotes

1 https://github.com/phuselab/pyVHR
